# Distinct Mechanisms of Imagery Differentially Influence Speech Perception

**DOI:** 10.1523/ENEURO.0261-19.2019

**Published:** 2019-09-13

**Authors:** Ou Ma (马欧), Xing Tian (田兴)

**Affiliations:** 1 Shanghai Key Laboratory of Brain Functional Genomics (Ministry of Education), School of Psychology and Cognitive Science, East China Normal University, Shanghai 200062, China; 2 NYU-ECNU Institute of Brain and Cognitive Science, New York University Shanghai, Shanghai 200062, China; 3 Division of Arts and Sciences, New York University Shanghai, Shanghai 200122, China

**Keywords:** efference copy/corollary discharge, internal forward model, memory retrieval, mental imagery, prediction, sensorimotor integration

## Abstract

Neural representation can be induced without external stimulation, such as in mental imagery. Our previous study found that imagined speaking and imagined hearing modulated perceptual neural responses in opposite directions, suggesting motor-to-sensory transformation and memory retrieval as two separate routes that induce auditory representation (Tian and Poeppel, 2013). We hypothesized that the precision of representation induced from different types of speech imagery led to different modulation effects. Specifically, we predicted that the one-to-one mapping between motor and sensory domains established during speech production would evoke a more precise auditory representation in imagined speaking than retrieving the same sounds from memory in imagined hearing. To test this hypothesis, we built the function of representational precision as the modulation of connection strength in a neural network model. The model fitted the magnetoencephalography (MEG) imagery repetition effects, and the best-fitting parameters showed sharper tuning after imagined speaking than imagined hearing, consistent with the representational precision hypothesis. Moreover, this model predicted that different types of speech imagery would affect perception differently. In an imagery-adaptation experiment, the categorization of /ba/-/da/ continuum from male and female human participants showed more positive shifts towards the preceding imagined syllable after imagined speaking than imagined hearing. These consistent simulation and behavioral results support our hypothesis that distinct mechanisms of speech imagery construct auditory representation with varying degrees of precision and differentially influence auditory perception. This study provides a mechanistic connection between neural-level activity and psychophysics that reveals the neural computation of mental imagery.

## Significance Statement

Our brain processes sensory information that we receive from the environment and mediates mental activity such as imagination. How do mental and perceptual processes interact to shape our cognition? We constructed a computational model that simulated how two types of imagery, imagined speaking and imagined hearing, differentially modulated perception via two distinct neural pathways. This model further predicted a choice shift in perceptual responses to ambiguous auditory stimuli, which was confirmed in a follow-up imagery-adaptation experiment. These results suggest that parallel neural pathways for mental imagery provide distinct functions to influence perception. These findings may implicate multiple strategies for constructing the brain-computer interface.

## Introduction

Perception results from interactions between bottom-up and top-down processes (Rao and Ballard, 1999; Hochstein and Ahissar, 2002; Friston and Kiebel, 2009; Schroeder et al., 2010; Rimmele et al., 2018). One of the top-down processes that influence perception is prediction, a process of simulating the environment and estimating the sensory input from possible future events (Bar, 2007; Szpunar et al., 2014; Keller and Mrsic-Flogel, 2018). However, where the prediction is generated in the brain and how the prediction influences bottom-up process to shape perception are still under debate (Aitchison and Lengyel, 2017).

Mental imagery has been hypothesized to be a predictive process (Moulton and Kosslyn, 2009; Tian and Poeppel, 2012). It has been used to investigate the origin and operations that mediate prediction (Tian and Poeppel, 2010, 2013, 2015; Tian et al., 2016, 2018). The predictive nature of imagery is manifested by inducing similar representation as perception without external stimulation (Kosslyn et al., 2001; Moulton and Kosslyn, 2009). The common generation of imagery is via memory retrieval (Kosslyn et al., 2001; Zatorre and Halpern, 2005; Hubbard, 2010; Tian and Poeppel, 2012). Because the movement of articulators causes speech, we proposed another stream for imagery, motor-based prediction, representations can be induced by simulating the planned motor commands and estimating their perceptual consequences (Tian and Poeppel, 2010, 2012).

The dual-stream for generating prediction has been supported by previous studies (Tian and Poeppel, 2013; Tian et al., 2016). In a magnetoencephalography (MEG) study (Tian and Poeppel, 2013), we used imagined speaking (articulation imagery, AI) or imagined hearing (hearing imagery, HI) to induce motor-based or memory-based prediction. We found that imagined speaking increased neural responses to the repeated auditory syllables, whereas imagined hearing caused suppression. The different directions indicate the distinct modulatory functions in the dual-stream prediction. However, the modulation effects cannot be easily explained without assuming parameters that operate in opposite ways, which contradicts the fact that two types of imagery should work similarly in nature.

**Figure 1.**
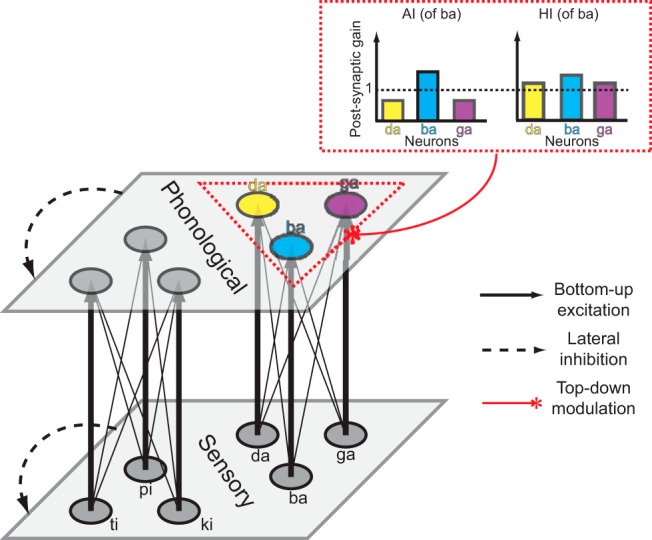
Illustration of the neural network model and hypothesis of the distinct top-down modulation effects from two types of speech imagery. The model contains two layers, the lower layer representing sensory (acoustic) processing and the upper layer the phonological analysis. Six nodes are included in each layer and are separated into two groups based on their phonemic features. Nodes within a group are fully connected across layers via excitatory connections (solid arrows), with more weights (thick) for the connections between nodes of the same syllable. Lateral inhibition (dashed arrow) is available among nodes in the same layer. The top-down modulation of speech imagery is modeled as changes of postsynaptic gain in a given group of neurons in the phonological layer (inserted dashed red box). The situation of imagined /ba/ in AI (articulation imagery) or HI (hearing imagery) was used as an example to illustrate the hypothesis. According to the hypothesis that a more precise representation would be activated via motor-to-sensory transformation, a more selectively boost of gain to the imagined syllable (increase of gain for /ba/ and decrease of gain for its neighbors) would occur in AI. Whereas in HI, the gains for neighbor syllables would also increase, making the gain for /ba/ relatively smaller.

To provide a parsimonious account, we hypothesized that the precision of induced representation was different (Tian and Poeppel, 2013; Tian et al., 2016). Because the articulatory movement uniquely defines the sound, the motor-based prediction in AI can be very precise. In contrast, because the memory-based prediction in HI could be noisy, the induced representation could be less precise, the neighboring representation could be activated, similar to the spreading activation model (Collins and Loftus, 1975; Anderson, 1983). Furthermore, the varying degrees of representational precision could differentially modulate the sensitivity to sounds and lead to distinct perceptual after-effects. The precise representation induced in imagined speaking can specifically increase the gain of repeated sound, whereas the relatively imprecise representation in imagined hearing can also increase the gain of neighboring sounds (Fig. 1, inserted red dashed box). The precise modulation reduces the lateral inhibition from neighbor neurons and yields repetition enhancement, whereas the increasing gain in all neighboring sounds causes more lateral inhibition and results in repetition suppression. That is, we hypothesized that different types of speech imagery would induce representation in distinct levels of precision and interact with the bottom-up process to shape perception differently.

To test this hypothesis, we built a neural network model with parameters of postsynaptic gain that were differentially modulated by two types of imagery (Fig. 1). The model was fitted to previous MEG results, and the best-fit parameters were obtained to test the hypothesis. Furthermore, the model predicted that two types of imagery would differentially shift the identification responses to ambiguous sounds (/ba/-/da/ continuum). We conducted a behavioral experiment to test how imagery influenced perception. Previous studies suggest that the degree of motor engagement constrains the precision of auditory representation (Oppenheim and Dell, 2008, 2010; Okada et al., 2018). The release from lip closure in bilabial stop /b/ is arguably more correlated with the downward jaw movement than the release from tongue closure in alveolar stop /d/. More efforts of movement inhibition in imagery /da/ than /ba/ would result in differential perceptual shifts. In summary, the aim of this study was to investigate the potential mechanisms that mediated mental imagery in the context of speech by testing how different types of mental imagery would modulate speech perception.

## Materials and Methods

We first provide an overview of our methods. A two-layer neural network model was built with free parameters (modulations of postsynaptic gain) that were differently tuned by two types of speech imagery. We used this model to fit our previous MEG data (Tian and Poeppel, 2013) and compared the best-fit values of the free parameters with our hypothesis. Next, we added a decision rule and fitted the free decisional parameters using the baseline (BL) behavioral results obtained from categorizing the /ba/-/da/ continuum sounds without the preceding imagery task. After fixing all free parameters, we derived a quantitative prediction on the shift of psychometric response curves to the /ba/-/da/ continuum after two types of speech imagery. We further conducted a behavioral experiment. The behavioral results of imagery-induced perceptual shifts were compared to the model prediction. In summary, the proposed model was established by fitting previous MEG neural data and was further tested in an independent behavioral experiment. In this way, this study provided strong evidence by connecting neural-level responses and psychophysics via a computational account to reveal how different types of speech imagery work and how they interact with, and shape, perception.

### Modeling

#### Basic unit and processing in the model

We built a two-layer neural network model to simulate the MEG adaptation results. The structure of this model was similar to the one in the published study (Huber and O'Reilly, 2003). Each node is a rate-coded unit with synaptic depression. This node is a simplified point neuron model, which can be viewed as a group of neurons that receive and output the same information, abstracting to a single mathematical point (O'Reilly and Munakata, 2000). Thereafter we use “neuron” or “node” interchangeably. The rate-coded output of a neuron, *o*, is the product of firing probability, *p*, and the postsynaptic firing amplitude, *a*, as in Equation 1:

(1) o=pa

The update of postsynaptic firing amplitude follows (Eq. 2):

(2) $$\frac{da}{dt}=R\left(1-a\right)-Do,$$

where the synaptic depression rate, *D* is driven by the output, *o*. The recovery rate, *R*, is governed by the current status of depression (1-*a*), so that the recovery term drives the firing amplitude, *a*, back to 1.

The firing probability, *p*, as in Equation 1, is determined by the differences between the membrane potential of a neuron, and the firing threshold, *θ*, as in Equation 3:

(3) p=v−θ.

Substitute Equation 3 into Equation 1, and the output is determined by

(4) o=(v−θ)a.

The membrane potential, *v*, updates according to Equation 5:

(5) $$\frac{dv\left(t\right)}{dt}=\tau \sum_{i}^{}C_{i}(E_{i}-v(t)),$$

where *τ* is a time constant indicating the speed of integration. The variables *C_i_*and *E_i_*represent the conductance and reversal potential of three channels, excitation, inhibition, and leak.

#### Model structure

In this two-layer neural network (Fig. 1), the first layer represents the acoustic analysis (hereafter termed the sensory layer, labeled using a superscript letter “*s*”), and the second layer provides a more abstract phonological analysis (hereafter the phonological layer, labeled using a superscript letter “*p*”). In each layer are six nodes representing the processing of different syllables. Based on the common features of syllables, three nodes were grouped together to represent syllables /ba/, /da/, and /ga/. The other group of three nodes represent syllables /pi/, /ti/, and /ki/. The nodes in the first and second layers within a group are fully connected. The connection strength value *w_ij_*(from neuron j to i) is 1 for the connections between the same syllable nodes in different layers, and 0.22 for connections between different syllable nodes. There are no connections between nodes across groups.

The signals from one neuron to another are obtained by multiplying the output, *o*, with the connection strength between the two neurons. These signals are summed over all input with other sources such as inhibition and leaking. By specifying the summation between and within layers and expanding from (Eq. 5), we obtained the complete updated equation for membrane potential of unit i at level *n* (Eq. 6):

(6) $$\frac{dv_{i}^{n}\left(t\right)}{dt}=\tau^{n}\left\{\left(1-v_{i}^{n}\right)\sum_{j}w_{ij}o_{j}^{n-1}-v_{i}^{n}\left[L+I\sum_{k}o_{k}^{n}\right]\right\}.$$

The member potential of neuron i at layer n, $v_{i}^{n}$, is updated according to the integration rate (time constant) of that layer $\left(\tau^{n}\right)$ summing over three sources of input. The first input is an excitatory input from the *n*–1 level via bottom-up connection strength *w_ij_*. The excitatory reversal potential *E_e_* is set to be 1, so that this bottom-up input drives the membrane potential to 1 (governed by the multiplier of 1-*v*). The second input is the leak with the fixed term *L.* The third input is the lateral inhibition, which is the strength of *I* multiplied by the sum of output t from *k* units at level *n*. The combination of the leak and lateral inhibition drive the membrane potential toward 0 because the reversal potential of *E_i_* and *E_l_* are set to 0 (as the term in the bracket is multiplied by -*v*). The fixed parameters are similar to those used in a previous study (Huber and O'Reilly, 2003) and listed in Table 1.

**Table 1.** Fixed parameters in the neural network model

Label	Description	Value
L	Leak strength	0.30
I	Inhibition strength	0.15
θ	Firing threshold	0.15
D	Depletion rate	0.324
R	Recovery rate	0.022
$\tau^{s}$	Time constant in the acoustic layer	0.031
$\tau^{p}$	Time constant in the phonological layer	0.01

#### Modeling top-down modulation

The top-down modulation effects of imagery were modeled as a gain parameter *g_i_* multiplying by the bottom-up input in the phonological layer of the nodes in the group of imagined syllable. Specifically, extending from Equation 6 by adding the gain parameter *g* that modulates the bottom-up input results in Equation 7:

(7) $$\frac{dv_{i}^{p}\left(t\right)}{dt}=\tau^{p}\left\{g_{i}\left(1-v_{i}^{p}\right)G_{A}\sum_{j}w_{ij}o_{j}^{a}-v_{i}^{n}\left[L+I\sum_{k}o_{k}^{p}\right]\right\}.$$

That is, according to the types of imagery and content, updating of the membrane potential of the node *i* at the phonological layer is subject to modulation of the gain parameter $g_{i}$ over the bottom-up input from the acoustic layer. These gain parameters can be considered as the sensitivity to one unit of total input from the lower layer. Based on the imagery type and imagery content, the modulation factor could increase the gain for the imagined syllable, but decrease or increase with smaller magnitude for the neighbor syllables; no effects over syllables were observed in the other group. The key assumption that distinct precision of representation in two top-down induction streams was modeled as the different modulation rates of postsynaptic gain (Fig. 1, inserted red dashed box). The AI task, because of the more precise representation of imagined syllable, would induce a positive shift in the gain for imagined syllables and a negative shift in gain for the neighbor syllables. In contrast, the HI task, because of noisier representation that led to activation of the representation of neighbor syllables, would induce a positive shift of the gain for all syllables in the group, but slightly more for the imagined syllable. The relatively greater boost of gain for the imagined syllable in AI would yield repetition enhancement as observed in our previous MEG study, whereas the relative smaller boost and positive shift of gain for the neighbor syllables in HI would induce more lateral inhibition and result in repetition suppression for the imagined syllable.

Moreover, the imagery tasks were assumed to increase attention to the subsequent sound, and different types of imagery may induce the attentional effect differently. Therefore, we model the attentional effect by adding another free parameter, $G_{A}$, which was applied to all neurons and raises the gain ubiquitously for all syllables. In total, there were six free parameters in two types, imagery modulation (*g_i_*, two for each imagery task) and attentional modulation (*G_A_*, one for each imagery task).

#### Fitting the modulation of gains with the MEG results

The free parameters of gain modulation in the model were fitted using the MEG results of imagery-induced repetition effects (Tian and Poeppel, 2013). The simulation was conducted in three stages. The first stage simulated the neural auditory responses to the syllables without repetition. During this stage, the free parameters of modulation gain were not included. The external stimuli were input to one of the neurons representing either /ba/ or /ki/, for 500 ms. The simulated neural responses were used as a BL and later subtracted from the responses after repetitions. Next, in the following two stages, free parameters of modulation gain were included, as in Equation 7, to simulate the neural responses after the AI and HI tasks. In the repeated condition of each task, the 500-ms duration external stimuli were provided as input to a given neuron in the first layer. The same group of neurons in the second layer was modulated by the gain parameters. In contrast, in the novel condition of each task, the gain modulation was applied to the group of neurons that did not contain the neuron receiving the external input in the first layer.

The output from all neurons at the phonological layer was summed to obtain the dynamics of simulated neural responses. Five waveforms were obtained, separately for auditory responses without repetition (BL), as well as responses after AI or HI in either repeated (AI repeated or HI repeated) or novel (AI novel or AI repeated) conditions. A temporally averaged measure was obtained in a 25-ms time window centered around the peak latency for each waveform. The simulation of repetition effects was calculated separately for the repeated and novel conditions in AI and HI, and it was quantified by the percentage change = (task-condition – BL)/BL. The six free parameters of imagery modulation and attentional modulation were fitted by minimizing the distance between the four simulated percentage changes and the empirical MEG results.

#### Generating prediction of imagery-biased perception to the /ba/-/da/ continuum

After fitting the MEG results, we fixed the free parameters of modulation gain. We further derived specific predictions concerning how the top-down processes could modulate behavior. A behavioral experiment of syllable identification was conducted to test the model prediction. In this experiment, participants were asked to identify syllables in the ambiguous auditory stimuli of /ba/-/da/ continuum, with or without performing a preceding imagined speaking or imagined hearing task (see the next section for the experimental procedure). The perceptual response changes as a function of imagery types were quantified and compared to the model prediction, hence further testing the mechanisms of differential gain modulation for different types of imagery tasks.

To link the simulated neural output with behavioral responses, a decision rule was applied as in Equation 8:

(8) $$p\left(c\right)=\frac{e^{N\left(t_{c^{\text{'}}}-t_{c}\right)}}{1+e^{N\left(t_{c^{\text{'}}}-t_{c}\right)}}+B_{c}.$$

That is, the percentage of choice, p(c), is the logistic function given by the differences between the peak latency of output from the neuron of the alternative syllable, $t_{c^{'}}$, and of the choice syllable, $t_{c}$, in the phonological layer. Because human scalp electrophysiology (EEG/MEG) reflects the accumulation of underlying neural activity over time, the peak latency of a waveform response indicates the time that an accumulator reaches a threshold. Therefore, we used the peak latencies from two competing nodes to quantify the proportion of choices. A decision noise parameter, *N*, was assumed to reduce the performance. Another parameter of decision bias toward the choice, $B_{c}$, was added to account for the overall shift across all levels of stimuli.

The two free parameters, decision noise, *N*, and bias, $B_{c}$, were fitted using the psychometric curve of responses to the /ba/-/da/ continuum. The first 100-ms input to the neurons in the sensory layer was determined by the physical differences among the levels of the /ba/-/da/ continuum stimuli. The first (most /ba/-like) and seventh (most /da/-like) stimuli had an input of 1 to /ba/ and /da/ neuron, respectively. For the fourth (middle, most ambiguous) stimulus, the input was set to 0.5 to both /ba/ and /da/ neurons. The input of the levels between the extreme and middle levels was determined proportionally according to the levels. That is, the second and third levels had inputs to the /ba/ neurons of 0.833 and 0.667 and to the /da/ neurons of 0.167 and 0.333, respectively. The fifth and sixth levels switched the proportion between the input to /ba/ and /da/ neurons to mirror those for the second and third levels. For the next 200 ms, the input represented the same vowel/a/, so that the input was 0.5 for both /ba/ and /da/ neurons.

The neural output waveform was obtained by running the simulation using the fixed parameters without the modulation gains in the neural network model. The peak latencies of output from the phonological layer /ba/ and /da/ neurons were identified for each level of input. The two free parameters, decision noise, *N*, and decision bias, $B_{c}$, were fitted by minimizing the distance between the simulated percentage of choice based on Equation 8 and the actual behavioral results of the psychometric curve of responses to the /ba/-/da/ continuum.

After fixing the free parameters in the decision rule, we generated the prediction of choice shifts to the /ba/-/da/ continuum after different types of imagery. The neural output waveform to different levels of the /ba/-/da/ continuum after either the AI or HI tasks was obtained by running the input of corresponding ambiguous levels through the neural network with fixed modulation gains for a given imagery task. The percentage of choice for each level in each task was obtained by the identified peak latencies using Equation 8 with the fixed parameters of decision noise, *N*, and decision bias, $B_{c}$. That is, the prediction of choice shifts after different types of imagery was generated without free parameters. This model prediction was compared with the behavioral results to test the model of modulation gain by different types of imagery.

### Behavioral experiment: imagery-induced perception shift and testing model prediction

#### Participants

Twenty-two participants were recruited from East China Normal University, and three of them were excluded. Two participants were excluded because of abnormal perceptual boundaries in the pretest (no differences among responses to different levels of /ba/-da/ continuum stimuli), and the third participant was excluded because this participant could not follow instructions and confused the AI task with the HI task (self-reporting of having difficulty separating two types of speech imagery). In total, nineteen participants were included in this study (10 females, average age of M = 22.58 and SD = 2.39). All participants were right-handed and received monetary incentives for their participation. This experiment was approved by the local institutional review board at New York University Shanghai. Written consent was obtained from each participant.

#### Materials

Pictures of a mouth and an ear were used as visual cues to indicate two different imagery conditions. Each image was presented foveally against a white background. The mouth picture indicated the AI condition and the ear the HI condition. One of two syllables, /ba/ and /da/, was placed at the center of the pictures to indicate the content of the imagery task in each condition.

**Figure 2.**
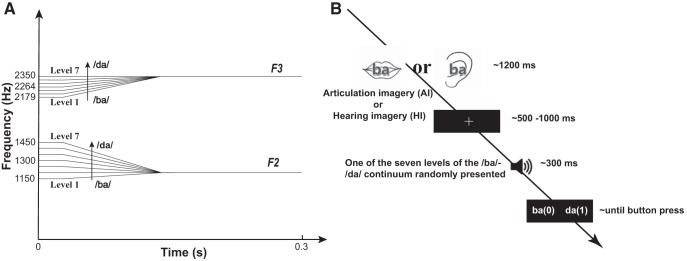
Auditory stimuli and experimental procedure. ***A***, Schematic plots of formant transition in each level of the /ba/-/da/ continuum. ***B***, Experimental procedure. A sample trial in the imagery conditions is depicted. At the beginning of the trial, a visual cue was presented for 1200 ms. The visual cue included either a picture of a mouth or an ear that indicated the following imagery tasks (AI or HI, respectively). A written syllable (either ba or da) was superimposed on the picture to indicate the content of the imagery tasks. Participants were asked to perform the imagery tasks during the duration of the visual cue presentation. After the offset of the visual cue, a fixation with a random duration ranging from 500 to 1000 ms appeared, followed by one of the seven levels of /ba/-/da/ continuum auditory stimuli. Participants were required to provide a perceptual judgment. The BL condition consisted of a similar procedure with the exception that no visual cue was presented and participants were not required to perform any imagery tasks before identifying the auditory syllable.

A seven-level /ba/-/da/ continuum, synthesized using Praat software (Boersma, 2002), was used as the auditory stimuli in this experiment (Fig. 2). Specifically, the onset frequency of F2 ranged from 1150 to 1450 Hz with an equal step of 50 Hz, whereas the onset frequency range of F3 was 2179–2350 Hz with a step of 28.5 Hz. The F1 was 750 Hz in all seven levels of stimuli. The stimuli were first created in a male voice with F0 of 120 Hz at 0.03 s, 130 Hz at 0.078 s, and 110 Hz at 0.27 s. Another set of stimuli in a female voice was created from the male voice stimuli using the function of “change gender” in the Pratt software. The formant shift ratio was set to 1.05, and the pitch median was set to 220. The female voice stimuli had a F0 of 218 Hz at 0.03 s, 236 Hz at 0.078 s, and 199 Hz at 0.27 s. The duration of all auditory stimuli was 300 ms. The intensity of stimuli was adjusted to a comfortable label for each participant, and it was kept consistent within each individual for all stimuli. The intensity ranged from 78- to 84-dB SPL across participants. These auditory stimuli were delivered through Sennheiser HD 280 Pro headphones. The experiment was conducted with Psychtoolbox in MATLAB (version 2016b).

#### Procedure

Three conditions were included in this experiment, AI, HI, and BL conditions. A sample trial is depicted in Figure 2*B*. In the imagery conditions (AI and HI), a visual cue (a picture of a mouth for AI and a picture of an ear for HI) was presented for 1200 ms. A written syllable, either a “ba” or “da,” was superimposed on the visual cue to indicate the speech content in the imagery tasks. During the duration of the visual cue presentation, participants were asked to imagine saying the indicated syllable in the AI condition or hearing someone else say that syllable to them in the HI condition. In the HI condition, participants were required to imagine hearing a voice that was opposite to their own gender. After the offset of the visual cue, a fixation with a duration ranging from 500 to 1000 ms appeared, followed by one of the seven levels of auditory stimuli. The gender of the auditory stimulus was also opposite to the gender of the participants’. A visual prompt indicating the perceptual judgment task followed. Participants were asked to judge whether the preceding sound was /ba/ or /da/ by pressing either key “0” or “1.” The mapping between choices and buttons was counterbalanced across participants. In the BL condition, a similar procedure was implemented with the exception that no visual cue was presented. Participants in the BL condition passively listened to the auditory stimuli and made a perceptual decision without any preceding imagery tasks.

Each condition was presented in a separate block. The block order was randomized. In each block, 252 trials were included with 36 trials for each of the seven levels of the /ba/-/da/ continuum stimuli. In the imagery conditions (AI and HI), participants were required to generate a mental imagery of /ba/ in half of the trials, and /da/ in another half. The presentation order of imagery content and auditory stimuli were randomized.

In the AI condition, participants were told to imagine saying /ba/ or /da/ without moving any articulators or producing any sounds. Instead, they were asked to generate the kinesthetic feeling of the articulator movement for a specific pronunciation and to generate the experience of their own voice loud and clear in their mind. In the HI condition, participants were asked to imagine hearing another person say the /ba/ or /da/ sound to them, while minimizing any kinesthetic feeling of movement. We did not obtain EMG recordings from around the larynx to objectively control for possible subvocalizations. Instead, we placed a microphone close to the participants’ mouth throughout the experiment and did not find any noticeable pronunciation. As long as no overt sound was produced and it was consistent across conditions, the perceptual changes observed in the study were not contaminated by the overt production.

To strengthen the memory process in HI, a familiarization phase was conducted only in the HI condition. Participants saw a picture of an average male or female face and listened to the /ba/ and /da/ sounds that were opposite to their gender three times each. Participants were told that the person on the screen produced those sounds. The familiarization phase was repeated after every 42 trials to help participants retain their memories during the HI phase. The opposite gender was used to better distinguish the internal voice in HI from their own voice. These manipulations aimed to selectively elicit the motor-induced auditory representation in AI and auditory memory in HI.

#### Analyses

Both response choice and reaction time (RT) were subject to a two-way repeated measures ANOVA with factors of task conditions (three levels) and sound levels (seven levels), followed by *post hoc* one-way repeated measures ANOVA with factors of task conditions at each sound level. To directly test the main hypothesis on the differential modulation effects of AI and HI, a two-way repeated measures ANOVA with factors of task conditions (two levels: AI and HI) and sound levels (seven levels) was conducted out. *Post hoc t* tests between imagery conditions were conducted at each level of sound.

χ^2^ Goodness-of-fit tests were conducted to statistically test the model fittings to the MEG repetition effects, the modeling fitting to the BL perceptual responses, and the similarity between the model predictions and behavioral results of imagery modulation.

### Code accessibility

The code described in the paper is freely available online at https://github.com/xtian0628/ImgMod.git. The stimuli, behavioral data, and code are available as Extended Data 1.

10.1523/ENEURO.0261-19.2019.ed1 Extended Data 1 Stimuli, behavioral data, and scripts. Download Extended Data 1, ZIP file.

## Results

### Fitting of top-down modulation MEG data

**Figure 3.**
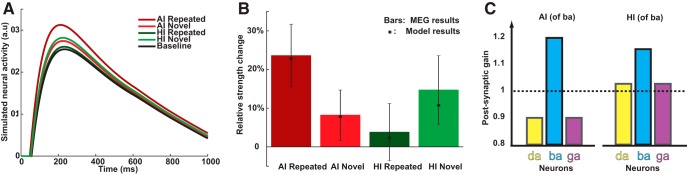
Simulation results of MEG imagery-induced repetition effects. ***A***, Model output of waveform responses. The simulated waveform responses in each condition have similar peak latencies of ∼200 ms after the auditory stimulus onset, consistent with the MEG results of observed effects in the M200 component (Tian and Poeppel, 2013). ***B***, Model simulation results of MEG imagery-induced repetition effects. The bar plots are repetition effects adapted from the MEG study (Tian and Poeppel, 2013). The stars represent the simulation results in each condition. The simulation captures the repetition enhancement in AI (repeated > novel) as well as the repetition suppression in HI (repeated < novel). ***C***, The values of the free parameters for best model fitting. The postsynaptic gain increases for the imagined syllable but decreases for neighbor syllables in AI, whereas the gain increases for all syllables for HI. These results of distinct gain modulation in different imagery tasks are consistent with the hypothesis in Figure 1.

The output from the phonological layer was obtained in different imagery conditions, as shown in Figure 3*A*. To further quantify the imagery-induced repetition effects, the temporal average responses around the peak latency of simulated neural activity was obtained in each condition. The response strength change (in percentage) relative to the BL response was calculated and compared to the empirical MEG results (Fig. 3*B*).

The model simulation results were fitted with the actual MEG results and captured the repetition enhancement and suppression in AI and HI, respectively (χ^2^ goodness of fit test, χ^2^(3) = 0.024, *p* > 0.99; the insignificance suggested no differences between the empirical and model results and hence a good model fit; Fig. 3*B*). Moreover, the parameters of gain modulation for the best-fit results (Fig. 3*C*) showed a similar profile as predicted (Fig. 1, inserted red dashed box). The postsynaptic gain increased for the imagined syllable but decreased for neighbor syllables in AI, whereas the gain increased for all syllables for HI. The best-fit values for all free parameters are listed in Table 2.

**Table 2.** Best-fit values of free parameters for the MEG repetition effects

Label	Description	Value
$g_{repeated,AI}$	Synaptic gain for the repeated syllable in *AI*	1.200
$g_{novel,AI}$	Synaptic gain for novel neighbor syllables in *AI*	0.899
$g_{repeated,HI}$	Synaptic gain for the repeated syllables in *HI*	1.157
$g_{novel,HI}$	Synaptic gain for novel neighbor syllables in *HI*	1.027
$G_{A,AI}$	Attentional gain in *AI*	1.088
$G_{A,HI}$	Attentional gain in *HI*	1.119

### Model fitting of behavioral responses to the /ba/-/da/ continuum and predictions of imagery modulation

**Figure 4.**
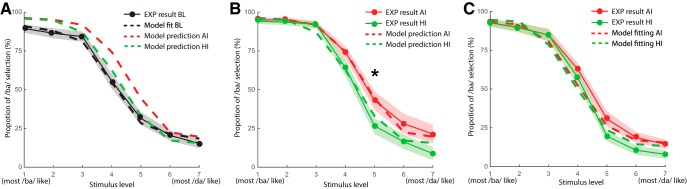
Model predictions and behavioral results. ***A***, Model fitting of the behavioral responses to the /ba/-/da/ continuum without imagery modulation and predictions of imagery modulation effects after imagery of /ba/. The psychometric curve of switching percepts from ba to da as a function of the stimulus level was obtained (solid black line and dots). The neural network model captured the behavioral responses by fitting two additional free parameters in the decision rule (the model fit is represented as the black dashed line). After fixing all free parameters, the model generated predictions about the imagery modulation after imagery of /ba/ (color dashed lines). Specifically, both types of imagery would have a positive shift in the psychometric curve, and the AI (red dashed line) would shift more than that of HI (green dashed line). ***B***, Behavioral results of imagery-induced modulation on the responses to the /ba/-/da/ continuum for the imagery content of /ba/. The psychometric curved in AI (solid red line and dots) showed a more positive shift than that of HI (solid green line and dots). The asterisk represent the significant difference between conditions in the experimental results at stimulus level 5 (*p* < 0.01). The model predictions (identical to those in ***A***) were superimposed and consistent with the observation. ***C***, Behavioral results after imagery of /da/ and model fitting. Behavioral results of imagery-induced modulation on the responses to the /ba/-/da/ continuum for the imagery content of /da/. The psychometric curved in AI (solid red line and dots) showed no significant difference from that of HI (solid green line and dots). The model fitted these results by adjusting the gain modulation (model fitting in dashed line). In each plot, the shaded areas around each line represented the ±SEM in each condition.

To generate predictions about behavioral responses from the neural network, we first examined the behavioral responses to the /ba/-/da/ continuum in the BL condition and fitted the decision rule using these behavioral responses without modulation of imagery. A psychometric curve was obtained for the categorization responses to the /ba/-/da/ continuum (Fig. 4*A*). A one-way repeated measures ANOVA revealed significant differences among the seven levels of /ba/-/da/ continuum auditory stimuli (*F*_(6,18)_ = 100.23, *p* < 0.001). These results indicated that a standard psychometric curve that reflected the perceptual categorization was obtained. Moreover, the model, after adding a decision rule, could fit the psychometric curve (χ^2^(6) = 1.38, *p* = 0.97; the insignificance suggested no differences between the empirical and model results and hence a good model fit; Fig. 4*A*). The best-fit values of free decisional parameters were decision noise, *N* = 0.0193, and decision bias to the choice, *B_c_* = 0.0469.

More importantly, by taking the neural output after imagery modulation, predictions of behavioral responses to the /ba/-/da/ continuum were generated. Specifically, the model predicted that the psychometric curve would shift upwards for both types of speech imagery, but the positive shift would be greater after imagined speaking (AI) than after imagined hearing (HI; Fig. 4*A*).

### Behavioral results of imagery modulation and comparison with model predictions

Next, we tested the predicted imagery modulation effects on perceptual categorization. Because motor involvement can affect the strength of the representation established in speech imagery (Oppenheim and Dell, 2008, 2010; Okada et al., 2018), the imagery of /ba/ and /da/ could have different degrees of modulation effects based on the number of articulatory movement needed to be inhibited during imagery, /ba/ for one (jaw movement) and /da/ for two (jaw and tongue moving). Therefore, the imagery of /ba/ could have stronger effects than that of /da/. First, we conducted a three-way repeated measures ANOVA with factors of stimulus-level, imagery task, and imagery token (ba or da). Significant main effects were found for all three factors, stimulus-level (*F*_(6,108)_ = 197.18, *p* < 0.001), imagery task (*F*_(1,18)_ = 10.65, *p* = 0.004), and imagery token (*F*_(1,18)_ = 13.61, *p* = 0.002). More importantly, the two-way interaction between the imagery token and imagery task was significant (*F*_(1,18)_ = 5.66, *p* = 0.029), as was the interaction between imagery task and stimulus-level (*F*_(6,108)_ = 3.53, *p* = 0.003). These significant interactions suggest that the content in the preceding imagery task greatly influenced the modulation effects of different types of imagery on perception. Therefore, we separated the imagery of /ba/ and /da/ in further analyses.

We first tested the modulation effects in the imagery tasks with the imagery content of /ba/. A two-way repeated measures ANOVA was conducted with two factors of task conditions (three levels) and sound levels (seven levels). The results showed significant main effects both for task conditions (*F*_(2,36)_ = 9.558, *p* < 0.001, η^2^ = 0.347) and sound levels (*F*_(6,108)_ = 169.963, *p* < 0.001, η^2^ = 0.904). More importantly, the interaction was also significant (*F*_(12,216)_ = 2.610, *p* = 0.003, η^2^ = 0.127). To assess the interaction, *post hoc* one-way repeated measures ANOVA was conducted among the task conditions for each level of auditory stimuli. Significant differences among task conditions were found at sound levels 2 (*F*_(2,18)_ = 4.676, *p* = 0.016), 3 (*F*_(2,18)_ = 4.808, *p* = 0.014), 4 (*F*_(2,18)_ = 8.154, *p* = 0.001), 5 (*F*_(2,18)_ = 6.250, *p* = 0.005), and 7 (*F*_(2,18)_ = 3.394, *p* = 0.045).

A similar two-way repeated measures ANOVA was conducted for the RT with two factors of task conditions (three levels) and sound levels (seven levels). The results showed that the main effect of sound levels was significant (*F*_(6,108)_ = 15.820, *p* < 0.001, η^2^ = 0.048), but not for the task conditions (*F*_(2,36)_ = 0.630, *p* = 0.538, η^2^ = 0.014). The interaction between them was significant (*F*_(12,216)_ = 1.954, *p* = 0.03, η^2^ = 0.009). A *post hoc* test did not reveal any significance among task conditions at any level of sound stimuli, suggesting that the RT for selection of choice in different conditions did not differ.

To directly test the model prediction on modulation differences between AI and HI (Fig. 4*B*), we conducted a two-way repeated measures ANOVA by including only the AI and HI conditions. The results revealed significant main effects both on task conditions (*F*_(1,18)_ = 14.13, *p* = 0.001, η^2^ = 0.01) and sound levels (*F*_(6,108)_ = 138.51, *p* < 0.001, η^2^ = 0.753). The interaction was also significant (*F*_(6,108)_ = 3.52, *p* = 0.003, η^2^ = 0.007). *Post hoc* comparisons showed significant differences between the AI and HI condition at sound level 5 (*t*_(19)_ = 4.236, *p =* 0.004). That is, participants who imagined speaking the syllable /ba/ had a higher chance of perceiving the following ambiguous stimuli as /ba/ than that when imagining hearing the same syllable. More importantly, the behavioral results of imagery adaptation were consistent with the model prediction (for AI, χ^2^(6) = 2.02, *p* = 0.92; for HI χ^2^(6) = 5.92, *p* = 0.43; Fig. 4*B*). The consistency between the model prediction and empirical results suggested that the varying precision of representation induced by the two types of imagery led to the distinct modulation of perceptual responses to ambiguous sounds.

A similar two-way repeated measures ANOVA was conducted for RT with two factors of task conditions (two levels, AI and HI) and sound levels (seven levels). The results showed a significant main effect of sound levels (*F*_(6,108)_ = 13.04, *p* < 0.001, η^2^ = 0.079), but neither the main effect of task conditions (*F*_(1,18)_ = 2.34, *p* = 0.144, η^2^ = 0.019) nor the significant interaction (*F*_(6,108)_ = 1.24, *p* = 0.292, η^2^ = 0.005), which suggested that the observed choice results could not be explained by the speed-accuracy trade-off.

When the content of imagery tasks was the syllable /da/, the results showed that the perception /ba/-/da/ continuum was not different among the AI, HI, and BL conditions (Fig. 4*C*), as the main effect of task conditions was not significant (*F*_(2,36)_ = 2.06, *p =* 0.142, η^2^ = 0.003). The main effect of sound level was still significant (*F*_(6,108)_ = 220.126, *p <* 0.001, η^2^ = 0.809), and there was a significant interaction between task conditions and sound levels (*F*_(12,216)_ = 1.924, *p* = 0.033, η^2^ = 0.005). However, *post hoc* tests did not reveal any reliable differences among conditions at any of the seven sound levels. For the RT results, only a main effect of sound levels (*F*_(6,108)_ = 5.527, *p* < 0.001, η^2^ = 0.023) was found. The main effect of task conditions (*F*_(2,36)_ = 0.519, *p* = 0.600, η^2^ = 0.0142) and the interaction (*F*_(12,216)_ = 0.851, *p* = 0.598, η^2^ = 0.004) were not significant.

We first used fixed parameters to fit the empirical results of imagery /da/. The χ^2^ goodness-of-fit test revealed significant differences between the model results and the empirical results (for AI, χ^2^(6) = 12.87, *p* = 0.045; for HI χ^2^(6) = 16.09, *p* = 0.013). These results suggested that the fitted parameters for imagery /ba/ could not automatically capture the imagery /da/ results, which was consistent with our hypothesis that the inhibition of different degrees of motor involvement in imagery /ba/ and /da/ influences the precision of induced representation.

Next, we fitted the empirical results of imagery /da/ by adding two free parameters, *Sg_i_* to scale the modulation gain and *SG_A_* to scale the attentional gain. The *Sg_i_* proportionally scales the gain for both imagined and neighboring syllables [Δ*g_i_* = – *Sg_i_* * (*g_i_* – 1)]. In this way, the tuning of the modulation can be changed. *SG_A_* is a multiplier that changes the attentional gain (*G_A_’* = SG_A_*G_A_). By scaling the modulation gain, the model can fit the behavioral results of imagery /da/ (for AI, χ^2^(6) = 4.483, *p* = 0.612; for HI χ^2^(6) = 7.034, *p* = 0.318; Fig. 4*C*). More importantly, the best-fit parameters were consistent with our hypothesis. For AI, the best-fit scaling parameters were *Sg_AI_* = 0.9056 and *SG_A_,_AI_* = 1.5328, whereas for HI, *Sg_Hi_* = 0.1063, *SG_A_,_HI_* = 1.4344. These results showed that the modulation gain for the imagery /da/ was scaled down from the fitted values of imagery /ba/. The scaling down was greater in the AI task than the HI task. These results were consistent with our hypothesis that imagery /da/ would induce a less precise representation because of the inhibition of greater motor involvement, compared to imagery /ba/.

## Discussion

A neural network model with a built-in gain modulation from different types of imagery successfully captured the different directions of repetition effects observed in a previous MEG study. The simulation results showed that the AI sharpened the gain more than HI. In the behavioral experiment of categorizing /ba/-/da/ continuum sounds, AI induced a greater choice shift in the preceding imagined syllable than HI. This positive perceptual shift was predicted by the model. Both the simulation and behavioral results were consistent with our hypothesis that motor-based speech imagery can induce a more precise auditory representation than memory-based imagery, which resulted in a differential influence on speech perception.

Our results provide evidence suggesting the origins and representational format of prediction. Mental imagery is assumed to be a predictive process by inducing representation without external stimulation (Moulton and Kosslyn, 2009; Tian and Poeppel, 2012). The model fitting results of different tuning in modulation gain suggest that predictions can be distinctively generated from motor and memory pathways. Moreover, more sharpened tuning in AI suggests a functional advantage of induction precision via the motor-based route.

More importantly, our results shed light on how the prediction operates and shapes perception. Previous studies of mental imagery have been mostly focused on the representational question, how similar are the representations induced by imagery compared to perception (Kosslyn et al., 1999; O'Craven and Kanwisher, 2000; Tian and Poeppel, 2010; Pearson and Kosslyn, 2015). This study, in contrast, extends to the computational question, what processes link imagery and perception. We hypothesized that prediction interacted with bottom-up processes via the modulation of gain along the perceptual hierarchy. Our proposed mechanisms are consistent with the theory from a Bayesian perspective (Aitchison and Lengyel, 2017). The prediction serves as a prior and modulates the likelihood generated from the bottom-up process to yield the posterior as results of perception. Our model and results agree with this integrative view that contrasts with the subtraction approach in the predictive coding (Friston, 2010) and active sensing (Morillon et al., 2015) theory, suggesting that the interaction between top-down and bottom-up processes may be mediated by multiple mechanisms.

The distinct behavioral results in conditions of different imagery content (/ba/ or /da/) further suggest that motor engagement constrains the precision of induced auditory representation. The pronunciation of /ba/ is physically realized by the movement of lips and jaw, whereas the articulation of /da/ involves the movement of tongue in addition to the jaw. The lip movement in /ba/ is arguably more correlated with the jaw movement, compared with the correlation between the jaw movement and the tongue movement in /da/. During imagery, one requirement is for the articulators to refrain from moving. Therefore, more efforts of movement inhibition would be in the imagery of /da/ compared with the imagery of /ba/. This greater inhibition of motor system during imagery of /da/ may induce a less precise representation than that of /ba/, and hence, a less positive shift was observed in the imagery of /da/. Our behavioral and simulation results (Fig. 4*B*,*C*) were consistent with this hypothesis as well as previous findings that motor involvement during imagery determines the precision and levels of induced representation (Oppenheim and Dell, 2008, 2010; Tian and Poeppel, 2013, 2015; Okada et al., 2018; Tian et al., 2018).

The model successfully explained the MEG results of distinct modulation directions caused by two types of imagery, which established the proposed mechanisms in a quantitative way. More importantly, it generated novel predictions that were more informative to test hypotheses than explaining existing data. After fixing all free parameters, we tested this model again by using a procedure that was orthogonal to the MEG experiment. We used new acoustic input to generate prediction at a new level (behavioral rather than neural level), and we tested this novel prediction using an independent dataset (distinct types of imagery modulate the psychometric curves of perception). In this way, our study provides strong evidence that supports a new computational account on how different types of speech imagery work and how they interact with and shape perception, the crucial aspect of computation in addition to the representation of mental imagery. Moreover, our study provides a mechanistic connection between neural-level and psychophysics, contrasting with previous studies on mental imagery.

The model trained on the neural data could predict the shifts in the psychometric curve after imagery /ba/. However, the fitted parameters could not reproduce the behavioral results in conditions of imagery /da/, suggesting that fitting the modulation in neural responses does not automatically lead to the results of the behavioral modulation. After adding a scaling parameter that adjusted the modulation gain, the model could fit the behavioral results of imagery /da/. The fitting results for imagery /da/ revealed that the modulation gain was scaled down compared with that under conditions of imagery /ba/. The scaling down was more prominent for the AI task compared with the HI task. These results suggest that in the AI task where motor-to-sensory transformation is required, the inhibition of motor involvement in the imagery task limits the precision of the induced representation. In contrast, in the HI task, which relies more on memory retrieval, the differences in motor involvement between syllables did not influence the precision of induced representation.

Attention could be a factor that mediates the observed perceptual shift differences between imagery tasks. However, two reasons make the attention less likely to be a confounding variable in our study. First, we informed participants that the auditory stimuli were randomly presented. There was no relation between what was imagined and the subsequent sound. They were asked to judge the sound solely based on what they heard. In contrast, a sound always followed the imagery task. Therefore, the potential attentional effect was more likely to be a temporal one, facilitating the initiation and processing of all sounds. We modeled the attention effect as a general modulation gain to all nodes. Second, an equal amount of attention would be induced in both imagined contents of /ba/ and /da/. However, we observed differences between the behavioral results when participants imagined different syllables, which highlights that motor involvement constrains the precision of the internally generated representation. Therefore, the observed results cannot be easily explained by different attentional levels between imagery tasks.

One of the difficulties in imagery studies is the lack of objective measures, especially in the auditory domain. In our previous studies, we attempted various designs, including a chronometric procedure (Tian and Poeppel, 2010) and repetition paradigms to “covert” the unobservable imagery to observable perceptual responses (Tian and Poeppel, 2013, 2015; Tian et al., 2018). The observed positive effects in the previous studies provide confidence that participants are engaged in imagery. Similarly, participants in this study were native to our hypothesis, yet we observed significant behavioral differences between conditions with different types of imagery, which suggests that the observed behavioral effects were most likely caused by imagery processes.

Our model simulation and behavioral results demonstrate that different types of mental imagery can distinctively modulate the neural and behavioral perceptual responses. This converging evidence suggests that mental and neural representation can be predicted and constructed internally without external stimulation via motor-based and memory-based pathways. The precision of internally constructed representation is determined by the nature of induction processes, a more precise representation can be induced by the motor-based process compared with the memory-based process. The differential precision levels of internally constructed representations can distinctively tune the sensory gain and shape perception.

## Synthesis

Reviewing Editor: Daniela Schiller, Mount Sinai School of Medicine Decisions are customarily a result of the Reviewing Editor and the peer reviewers coming together and discussing their recommendations until a consensus is reached. When revisions are invited, a fact-based synthesis statement explaining their decision and outlining what is needed to prepare a revision will be listed below. The following reviewer(s) agreed to reveal their identity: Edmund Lalor. Note: If this manuscript was transferred from JNeurosci and a decision was made to accept the manuscript without peer review, a brief statement to this effect will instead be what is listed below.

This is an interesting study on the important topic of neural representations of imagined speech. Specifically, the idea that different types of imagery can affect perception via different mechanisms. The authors present and test a model that predicts effects of imagined hearing vs speaking speech, nicely linking computational models to neural data and to behavior.

Many thanks indeed to the authors for their efforts in responding to the previous reviews. They have done a very thorough job in their rebuttal and have satisfied most concerns. The revised manuscript is a further improvement over the previous submission also with regard to spelling/grammar, which is now acceptable.

Action required:

The absence of an objective control for subvocalization effects (e.g. EMG recordings from around the larynx) during the behavioral task is a limitation of the study. Having a microphone is not sufficient to rule out these effects in AI and HI condition. The authors should highlight the subvocalizations issue as a limitation.

Other minor comments:

Keywords:

l. 55: “speech perception” is already in the title and thus is redundant as a keyword.

Results:

l.446, l.462: “p>0.99”, “p=0.97” is an off way reporting p-values; it should instead be “p<0.01” and “p=0.03”, respectively.

## Author Response

Dear Dr. Daniela Schiller,

We thank you and reviewers again for your constructive comments. We are pleased to see that we have addressed most reviewers' concerns. In this round of revisions, we have updated our manuscript to provide further clarifications. Please find our point-by-point responses in bold following each comment below.

The absence of an objective control for subvocalization effects (e.g. EMG recordings from around the larynx) during the behavioral task is a limitation of the study. Having a microphone is not sufficient to rule out these effects in AI and HI condition. The authors should highlight the subvocalizations issue as a limitation.

We have highlighted this subvocalization issue in the Method section.

['We did not obtain EMG recordings from around the larynx to objectively control for possible subvocalizations.']

Other minor comments:

Keywords:

l. 55: “speech perception” is already in the title and thus is redundant as a keyword.

The redundant keyword has been deleted.

Results:

l.446, l.462: “p>0.99”, “p=0.97” is an off way reporting p-values; it should instead be “p<0.01” and “p=0.03”, respectively.

We implemented the chi-square goodness-of-fit test to exam whether the model can fit the empirical data. The insignificant results (p > 0.05) suggested that the empirical data and model simulations were not different - failed to reject the null hypothesis, and hence supported a good model fit. We have provided clarifications after these results.

['The insignificance suggested no differences between the empirical and model results and hence a good model fit.']
