# Supplementary material for: Distinct Mechanisms of Imagery Differentially Influence Speech Perception
Source: eNeuro. 2019 Sep 13;6(5):ENEURO.0261-19.2019. doi: 10.1523/ENEURO.0261-19.2019 (PMC6753248; doi:10.1523/ENEURO.0261-19.2019)
Supplement: Extended Data 1 — Stimuli, behavioral data, and scripts. Download Extended Data 1, ZIP file. [file sup_enu-eN-NWR-0261-19-s02.zip › stim_beh_exp/bada_generating_scripts_Praat.doc]

\# the steady state formant values

f1 = 750

b1 = 100

f2 = 1200

b2 = 150

f3 = 2350

b3 = 200

f4 = 3300

b4 = 300

f5 = 4000

b5 = 500

f6 = 4600

b6 = 600

#the onset formant values

f1bd = 300

f2b = 1100

f2d = 1600-150

f3b = 2150

f3d = 2500-150

Create PitchTier... empty 0 0.25*(6/5)

Add point... 0.024*(6/5) 0

Add point... 0.025*(6/5) 120

Add point... 0.065*(6/5) 130

Add point... 0.225*(6/5) 110

Add point... 0.226*(6/5) 0

To Sound (pulse train)... 22050 1 0.05 2000 no

Rename... source

for i from 1 to 7

#calculate onset values for f2 and f3

for f from 2 to 3

barkOnset = (7-i)/7 * hertzToBark(f'f'b)+ i/7 * hertzToBark(f'f'd)

f'f'Onset = barkToHertz(barkOnset)

endfor

Create FormantTier... bda'i' 0 0.25*(6/5)

Add point... 0.025*(6/5) 'f1bd' 'b1' 'f2Onset' 'b2' 'f3Onset' 'b3' 'f4' 'b4' 'f5' 'b5' 'f6' 'b6'

Add point... 0.075*(6/5) 'f1' 'b1' 'f2' 'b2' 'f3' 'b3' 'f4' 'b4' 'f5' 'b5' 'f6' 'b6'

Speckle... 0 0 5500 30 yes

plus Sound source

Filter

Rename... bada'i'

endfor
